# A healthy lifestyle text message intervention for adolescents: protocol for the Health4Me randomized controlled trial

**DOI:** 10.1186/s12889-022-14183-9

**Published:** 2022-09-23

**Authors:** Rebecca Raeside, Karen Spielman, Sarah Maguire, Seema Mihrshahi, Katharine Steinbeck, Melissa Kang, Liliana Laranjo, Karice Hyun, Julie Redfern, Stephanie R. Partridge, the Health4Me Team, Maree L. Hackett, Gemma Figtree, Robyn Gallagher, Kyra A. Sim, Tim Usherwood, Charlotte Hepse, John Skinner, Katrina E. Champion, Lauren A. Gardner, Kathryn Williams, Danielle Castles

**Affiliations:** 1 Engagement and Co-Design Research Hub, School of Health Sciences, Faculty of Medicine and Health, University of Sydney, Sydney, NSW Australia; 2 InsideOut Institute, Faculty of Medicine and Health, University of Sydney, Sydney, NSW Australia; 3 Sydney Local Health District, Sydney, NSW Australia; 4 Department of Health Sciences, Faculty of Medicine, Health and Human Sciences, Macquarie University, Sydney, NSW Australia; 5 Department of Adolescent Medicine, The Children’s Hospital at Westmead, Sydney, NSW Australia; 6 Specialty of Child and Adolescent Health, Westmead Clinical School, Faculty of Medicine and Health, University of Sydney, Sydney, NSW Australia; 7 General Practice Clinical School, Sydney Medical School, Faculty of Medicine and Health, University of Sydney, Sydney, NSW Australia; 8 Westmead Applied Research Centre, Faculty of Medicine and Health, University of Sydney, Sydney, NSW Australia; 9 Western Sydney Primary Health Network, Sydney, NSW Australia; 10 Department of Cardiology, Concord Repatriation General Hospital, ANZAC Research Institute, Sydney, NSW Australia; 11 The George Institute for Global Health, University of New South Wales, Sydney, NSW Australia; 12 Prevention Research Collaboration, Faculty of Medicine and Health, University of Sydney, Sydney, NSW Australia

**Keywords:** Adolescent, Prevention, Physical activity, Nutrition, mHealth, Text message, Randomized controlled trial

## Abstract

**Background:**

Adolescence presents a window of opportunity to establish good nutrition and physical activity behaviours to carry throughout the life course. Adolescents are at risk of developing cardiovascular and other chronic diseases due to poor the complex interplay of physical and mental health lifestyle risk factors. Text messaging is adolescents main form of everyday communication and text message programs offer a potential solution for support and improvement of lifestyle health behaviours. The primary aim of this study is to determine effectiveness of the Health4Me text message program to improve adolescent’s physical activity or nutrition behaviours among adolescents over 6-months, compared to usual care.

**Methods:**

Health4Me is a virtual, two-arm, single-blind randomised controlled trial, delivering a 6-month healthy lifestyle text message program with optional health counselling. Recruitment will be through digital advertising and primary care services. In total, 390 adolescents will be randomised 1:1 to intervention or control (usual care) groups. The intervention group will receive 4–5 text messages per week for 6-months. All text messages have been co-designed with adolescents. Messages promote a healthy lifestyle by providing practical information, health tips, motivation and support for behaviour change for physical activity, nutrition, mental health, body image, popular digital media and climate and planetary health. Virtual assessments will occur at baseline and 6-months assessing physical health (physical activity, nutrition, body mass index, sleep), mental health (quality of life, self-efficacy, psychological distress, anxiety, depression, eating disorder risk) and lifestyle outcomes (food insecurity and eHealth literacy).

**Discussion:**

This study will determine the effectiveness of a 6-month healthy lifestyle text message intervention to improve physical activity and nutrition outcomes in adolescents.

**Trial registration:**

Australia New Zealand Clinical Trials Registry (ANZCTR) ACTRN12622000949785, Date registered: 05/07/2022.

## Introduction

Adolescence presents a window of opportunity to establish good nutrition and physical activity behaviours, which are the pillars of overall future physical and mental health [1]. However, the current picture of adolescent physical and mental health worldwide is concerning. Research has established associations between several lifestyle risk factors, including physical activity, nutrition, sedentary behaviour and sleep that are contributing to the declining physical and mental health status of adolescents [2, 3]. Adolescents in high income countries, like Australia are not meeting national guidelines for these risk factors. National Australian data from 2017 to 18 and 2011–12 found 96% of adolescents do not meet national guidelines for fruit and vegetable intake [4], and 41% of their total energy intake came from discretionary foods [5], respectively. Globally more than 80% of adolescents are not engaged in sufficient physical activity [6]. Reducing sedentary behaviour is important for both physical and mental health. However, Australian data from 2017 to 18 found only 7.9% of 13–17-year olds met the physical activity guideline, and only 20% met the sedentary screen-based behaviour guideline [7]. Short sleep duration amongst adolescents is also prevalent. For example, in a sample of American students, 6 out of 10 middle school students and 7 out of 10 high school students were not sleeping for the recommended 8–10 hours per night [8]. The COVID-19 pandemic negatively impacted the lifestyle behaviours Australian adolescents. Cross-sectional data from a sample of 983 adolescents indicated increases of excessive recreational screen time (86 to 94%) and insufficient fruit intake (20 to 30%). As well, this data indicated proportions of adolescents engaging in insufficient physical activity (82%) and vegetable intake (84%) remained concerning [3]. The intersection of risk factors for both obesity and mental health demonstrates the need for holistic programs which provide support to improve lifestyle risk factors.

Systematic reviews have shown that poor nutrition, physical inactivity, sedentary behaviour and sleep are all linked to poor mental health [9–11] and to obesity [12, 13]. Globally, the presence of overweight and obesity among children and adolescents 5–19 years has risen from 4% in 1975 to over 18% in 2016 [14]. By 2030 it is predicted there will be 254 million children and adolescents with obesity [15]. National Australian data from 2017 to 18 found 25% of children and adolescents 2–17 years have overweight or obesity [16]. Similarly, the period of adolescence is the highest risk developmental stage for the onset of mental illness [17]. In adolescents it has been shown that there is a bi-directional association between overweight and obesity and poor mental health [18, 19]. Overweight and obesity during adolescence has immediate impacts on quality of life [20], which if not addressed may have long term consequences on wellbeing [21]. Research has also demonstrated significant links of unhealthy diets to mental health in adolescence [9, 22]. Adolescent mental health is of growing concern with 14% of 10–19-year-olds globally experiencing a mental disorder with depression, anxiety and behavioural disorders among the leading causes of illness and disability [1]. In Australia, 20% of adolescents aged 12–17 had high or very high levels of psychological distress and 14% experienced a mental disorder [23]. The COVID-19 pandemic has also significantly disrupted the lives of Australian adolescents, with research showing a worsening in mental health [24], with the potential for long-term impacts [25]. Failing to address mental health conditions in adolescence can harm physical and mental health and hinder leading a fulfilling life in adulthood [1], displaying a growing need for action in these areas.

The current approach to improving the health behaviours of adolescents needs to be aligned with best practice recommendations [26]. Programs that target mental health often do not focus on nutrition and physical activity behaviours [27] and programs with an obesity prevention or management lens can be stigmatizing [28]. There is a need to develop accessible and scalable programs which focus on prevention through management of lifestyle health behaviours and mindful of impacts on mental health. This is in line with the Australian National Obesity Strategy 2022–32 where young people recognised the need for holistic approaches to support a healthy lifestyle [29]. Recent research has shown that the primary care health setting in Australia is appropriate for delivering preventive care to adolescents [30]. However, the barriers to primary care services are bi-directional, adolescents cite barries including cost of visits, opening hours, transport to and from services, concerns about confidentiality and feeling embarrassed [31]. General practitioners (GPs) have indicated that time is a major barrier as adolescents often need longer consultations to build strong relationships and overcome communication barriers to deliver lifestyle health information [30]. Therefore, for prevention programs to be effective, these barriers must also be addressed so that adolescents are empowered to improve their lifestyle health behaviours.

The ubiquitous nature of mobile health (mHealth) programs offers a potential solution to address these risks. There is strong evidence supporting the efficacy of text messaging in adults for facilitating positive behaviour change for physical activity [32], nutrition [33], mental health [34] and management of cardiovascular disease risk [35]. A systematic review assessing text messaging as an intervention for adolescent physical activity and sedentary behaviour found that there was high heterogeneity of study design which prevented conclusions as to which intervention elements were linked to increased effectiveness, and therefore concluded that further research is needed with text messaging as the focus of the intervention to demonstrate the effect of text messages on these lifestyle health behaviours [36]. Text messaging is well suited to adolescents as 94% of adolescents in Australia own a mobile phone [37], text messaging is their main form of communication and direct communication can be made with participants for minimal cost. Text messaging also improves accessibility to programs from those in rural, remote, and disadvantaged populations who have inequitable access to healthcare and where the COVID-19 pandemic has only increased these inequities [38]. There is a need for simple and accessible solutions that focus on prevention, specifically targeting physical and mental health among adolescents.

The primary aim of this study is to determine, in a single blind randomized controlled trial (RCT), whether a semi-personalised text message healthy lifestyle program (Health4Me) compared to usual care will improve adolescents’ physical activity (moderate to vigorous physical activity [MVPA] minutes per day) or nutrition (vegetable intake > 3 serves per day) behaviours among adolescents over 6-months. Further, to determine if the text message program can improve or maintain other physical and mental health and lifestyle outcome, to explore the acceptability, utility and engagement with Health4Me among youth and inform implementation into primary health care services in Australia.

## Methods

### Study design

**Fig. 1:**
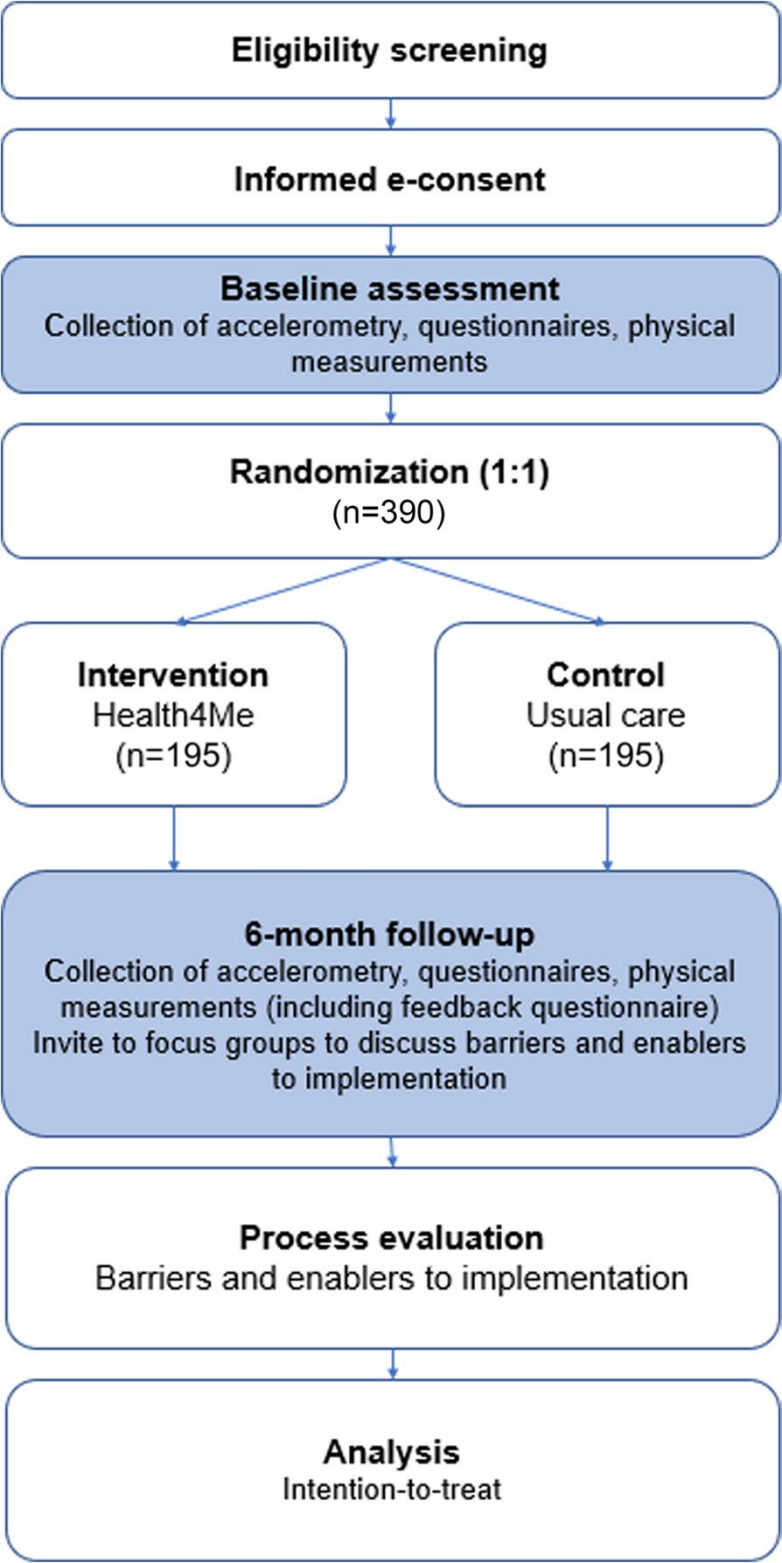
Flow chart of study design for Health4Me study

The Health4Me study is a virtual, randomised, controlled, single-blind trial, which delivers a 6-month text message healthy lifestyle program with optional health counselling to 390 healthy adolescents (12–18 years old inclusive) (Fig. 1).

### Randomization and blinding

Participants will be randomly allocated to either the intervention or control group. The control group will receive usual care (no intervention). Participants will view digital advertisements for the study on social media, after clicking on a link it will take them to the participant information sheet which they can read and provide informed e-consent. After obtaining electronic consent from adolescents (and their parents or guardians if < 14 years old) and completing the baseline assessment on a secure web-based database, a member of the research team will randomize the participant via a centralized, computerized randomisation program in a uniform 1:1 allocation ratio (intervention: control). Randomization is based on both permuted blocks with randomized block sizes and stratification, where the strata are age and gender. A randomization list will be generated by an independent statistician. Members of the research team who enrol and conduct follow-ups with participants will be blinded to group allocation.

For each participant, the randomization program automatically produces a study identification number. On the following Monday after the baseline assessment, the computer system automatically sends the assigned text message program to the participant. Therefore, the random allocation sequence will be concealed from study personnel at baseline and at 6-months. Participants will be encouraged not to discuss whether they are or are not receiving the text messages until the end of the 6-month follow-up.

### Study population

As the study is virtual, there are no physical sites for recruitment. Rather, participants will be recruited through digital advertisements. GPs will also be informed of the study via email and encouraged to advertise this to their adolescent patients. A log of all recruitment strategies will be kept. Participants will be eligible to take part if they are: (1) 12–18 years old inclusive; (2) own a mobile phone capable of sending and receiving text messages; (3) have sufficient English to read text messages pitched at a 7th grade reading level; and (4) provide electronic consent (or from their parents or guardians if < 14 years old).

Participants will be excluded if they: (1) have a diagnosis of type 1 or type 2 diabetes mellitus; (2) have a previous or current diagnosis of an eating disorder or are at high risk for an eating disorder as assessed in screening; (3) weight < 25th centile for age; (4) have had recent rapid weight loss; (5) have a medical condition which would preclude providing informed consent or ability to comply with the study protocol; (6) are enrolled in an alternative randomised lifestyle management program; (7) are pregnant or planning to become pregnant during the 6-month intervention; and (8) are unable to read English at a 7th grade reading level. Screening will occur for eating disorders at baseline, 2, 4 and 6-months and any participant who scores above the pre-determined threshold will trigger the study team to refer appropriately. Both screening and recruitment logs will be kept by the research team for those participants who are ineligible or decline to participate, including reasons for non-participation.

### Control group

The control group will receive usual care. For this study, usual care is defined as accessing available information and health services to maintain a healthy lifestyle. Participants in the control group receive an initial text message welcoming them to the study, and one text message at 2, 4 and 6 months respectively. The messages at 2 and 4 months will have a link to complete the eating disorder screening and the message at 6 months will direct them to complete all follow-up assessments. Participants in the control group will be offered the text message intervention after the 6-month follow-up assessments are complete at no cost. However, they will not be able to receive health counselling.

### Intervention group

The intervention group will receive usual care, plus a 6-month text message support program with text messages focussed over six key priority areas for improving physical and mental health, namely nutrition, physical activity, mental health, body image, climate and planetary health and popular digital media (e.g., social media), as well as the option to speak with a university-trained health counsellor.

#### Message content

Message program content was developed according to a previously published model [39]. Briefly, an established youth advisory group (comprised of 16 adolescents aged 13 to 18 years old inclusive) identified current top health issues for young people [40] and assisted in drafting relevant and practical content, informed by national physical activity, dietary and sedentary screen-based behaviour guidelines, behaviour change theories and lived experience of being a young person in today’s world. Furthermore, text messages will also include information on how the primary care system works and how to connect with primary care services, including GPs. Included in the bank of text messages is one welcome message and one final text message. Each message will have a unique signature as the study name to ensure that participants know these messages are from the research study and participants at enrolment will be told how to unsubscribe if required.

#### Message frequency and sequence

Each intervention participant will receive a semipersonalized and customized set of text messages, sent on four random days per week and at random times. If the participant is attending high school, the weekday text messages will only be sent before or after school hours (8.00 AM to 9.00 AM or 3.30 PM to 7.30 PM). Each text message is unique and will only be sent once throughout the 6-month program. The messages are semipersonalized by including the participants name and selecting content relevant to their age and dietary behaviours (e.g., vegetarian). Participants can also update their key information, (e.g., change in dietary behaviours) through the course of the study and this will be reflected in the ongoing text messages they receive. Messages are sent at no cost to the participants and a bulk-rate cost to the study. However, if participants reply to the study team, these will be paid for by the participant at standard short message service (SMS) rates. The intervention will encourage two-way communication; however, it is for the participant to decide how much they engage with the study. All replies and responses are monitored by the health counsellor and responses given in 72 hours when required.

#### Health counsellor

Once a month (6 in total), intervention participants will be sent a text message encouraging them to call the university qualified health counsellor to ask questions or request additional information. The personalised health counselling calls will last 10–15 minutes and will be delivered according to standardized protocol. The university qualified health counsellor (allied health or public health professional) will monitor and respond to participants’ request for a call each month either via text message or phone call within 3 working days. Participants are allowed 6 health counselling calls in total over 6-months. The health counselling calls will allow participants to set behavioural goals, discuss barriers and enablers to behaviour change, and their overall progress. This part of the intervention is based on the evidence based TEXTBITES Study for obesity prevention in adolescents [41].

#### Text message management system

The text message management system has been used successfully in two randomised controlled trials (TEXTBITES and EMPOWER-SMS) [42, 43]. Pre-specified algorithms are put in place to ensure that the intervention group are the only ones who receive the Health4Me message program. Each week, messages are randomly selected from the text message bank by the software system such that a variety of messages are delivered each week from the four key priority areas. Both groups are sent a welcome message at the beginning of the study and a concluding message after 6-months. Participants are instructed in the welcome message to save the dedicated number that the text messages come from, so that they are not seen as spam. They are also given details on how to unsubscribe from the messages if required. All participants receive information on safe and acceptable times to read text messages (e.g. not to read messages while driving) and contact details of the research team in case of any issues. A member of the research team will manage a study mobile phone, and a record is kept of all incoming messages from participants and outgoing messages from the health counsellor. Analysis of incoming and outgoing messages will be performed at the end of the study, as part of the process evaluation. If a participant from either group wishes to withdraw from the study, they can at any time by replying ‘STOP’ to any of the messages or contacting the research team. If a reason for withdrawal is provided, it will be recorded in the enrolment log.

### Data collection and study outcomes

Data will be collected from participants at baseline and 6-months online. In addition, for safety the eating disorder screening will be conducted at 2-months and 4-months for all participants. The co-primary outcomes, secondary outcomes and their assessments are provided below in Table 1. Multiple endpoints have been selected such that a significant effect against either one may be taken as evidence of efficacy. The co-primary outcomes are change in moderate-to-vigorous minutes of physical activity (MVPA) minutes per day and change in the proportion of participants meeting vegetable intake guidelines (> 3 serves per day). MVPA will be measured by ActivInsights Geneactiv wrist-worn accelerometers [46]. After enrolment and at the 6-month assessment, participants will be mailed an accelerometer to wear for 7 days and then return by pre-paid post to the research team. Vegetable intake will be measured using data from the Australian Child and Adolescent Eating Survey (ACAES), developed by the University of Newcastle [44].

**Table 1:** Description of Health4Me study outcomes and assessments

Outcome	Assessment
*Dual Primary Outcomes*	
Vegetable intake	Australian Child and Adolescent Eating Survey [ACAES] [44, 45]
Moderate-vigorous physical activity (MVPA) min/day	Geneactiv activity and sleep unit worn for 7 days [46]
*Secondary Outcomes*	
BMI^a^ z-score	Units BMI is above or below average for the age- and sex-specific reference values, using participant self-reported weight and height [47]
Waist-to-height ratio	Mid measure between the iliac crest and lowest rib and height, using participant self-reported waist circumference and height [47]
Dietary quality	Australian Child and Adolescent Eating Survey [ACAES] [44, 45]
Sleep quality	Pittsburgh Sleep Quality Index Short [PSQI-Short] [48, 49]
Health-related quality of life	Child Health Utility instrument [CHU9D] [50]
Self-efficacy	Self-efficacy for Healthy Eating and Physical Activity [SE-HEPA] [51]
Anxiety	General Anxiety Disorder-7 [GAD-7] [52]
Depression	Centre for Epidemiological Studies Depression Scale Revised-10 [CESDR-10] [53]
Psychological distress	Kessler Psychological Distress Scale [K6] [54]
Eating disorder risk	InsideOut Institute screening tool [IOI-S] [55]
Food insecurity	USDA^b^ 6-item food security module [56]
eHealth literacy	eHEALS eHealth Literacy Scale [57]

^a^*BMI* Body mass index

^b^*USDA* United States Department of Agriculture

Weight, height and waist circumference will be self-reported by participants according to a standardised digital protocol [47]. Participants will be shown how to correctly measure weight, height and waist circumference by videos put together by the research team and embedded into the data collection form. Self-reported height and weight has been validated amongst young adults in an Australian population [58–60]. Weight, height and waist circumference measurements will be used to calculate BMI z-score (using age and sex specific reference values) and waist-to-height ratio.

The following questionnaire-based assessments have demonstrated reliability and validity in adolescent populations and will be completed online at baseline and follow-up assessments. Sleep quality will be measured using the Pittsburgh Sleep Quality Index-Short [48, 49]. Health related quality of life and self-efficacy will be measured by the Child Health Utility Instrument (CHU9D) [50] and Self Efficacy for Healthy Eating and Physical Activity (SE-HEPA) [51] respectively. Anxiety, Depression and Psychological Distress will be measured by the General Anxiety Disorder-7 (GAD-7) [52], Centre for Epidemiological Studies Depression Scale Revised-10 (CESDR-10) [53] and the Kessler Psychological Distress Scale (K6) [54]. Eating disorder risk will be measured using the InsideOut Institute screening tool (IOI-S) [55]. Food insecurity will be measured with the USDA 6-item food security module [56] and eHealth literacy with eHEALS eHealth Literacy Scale [57]. A schedule of enrolment, interventions and assessments is presented in Table 2. All data will be entered electronically into Research Electronic Data Capture (REDCap), hosted on secure servers by the University of Sydney. Electronic data will be monitored by the research team through monthly reports to ensure quality and completeness of the data.

**Table 2:** Health4Me study schedule of enrolment and assessments

Assessments	Pre-baseline	Baseline	6-month follow-up
**Enrolment**			
Eligibility screen	✓		
Informed consent	✓		
Randomisation		✓	
**Assessments**			
Demographics		✓	
Physical activity		✓	✓
Dietary intake		✓	✓
BMI^a^ z-score		✓	✓
Waist-to-height ratio		✓	✓
Sleep quality		✓	✓
Quality of life		✓	✓
Self-efficacy		✓	✓
Anxiety		✓	✓
Depression		✓	✓
Psychological distress		✓	✓
Eating disorder risk		✓	✓
Food insecurity		✓	✓
eHealth literacy		✓	✓
Process evaluation			✓

^a^*BMI* Body mass index

### Process measures

The acceptability, utility and engagement with the Health4Me Program will be measured through quantitative and qualitative measures. Firstly, text message data will be extracted from the software system to assess engagement by the number of messages sent, number of responses received, and number of messages ‘bounced’ and undelivered. Secondly, a study-specific user feedback and satisfaction questionnaire will be administered to all participants. It will contain questions on a 5-point Likert scale from strongly agree to strongly disagree regarding acceptability and utility of the program. It will also include open ended questions regarding most and least useful program components and suggestions for program improvement. These responses will be coded thematically, and emerging themes identified. The questionnaire will also ask questions about whether participants used or accessed any other digital health tools to help manage their lifestyle health behaviours during the study. Intervention participants will be invited by text message, email, or phone call to join focus groups after the 6-month follow-up. A minimum of five focus groups will be conducted consecutively until thematic saturation is reached. The focus groups will be conducted via Zoom teleconference and last approximately 45 minutes. Participants will be purposively selected to ensure a mix in terms of age, ethnicity and location are represented to encompass different viewpoints.

### Statistical considerations

For the outcome of MVPA (minutes per day), 312 (156 per group) participants are needed to achieve 90% power to observe the mean difference of 14.8 (control: 42.55 and intervention: 57.36) with standard deviations (SD) of 21.45 for control and 37.79 for intervention and accounting for 30% dropout. A two-sided two-sample unequal-variance t-test was used. The Bonferroni adjusted significance level of 0.025 was used to account for two primary outcomes. To detect a difference in vegetable intake at 6-months, 390 (195 per group) participants will achieve 90% power to detect 13.37% difference in the proportion of appropriate vegetable consumption (control: 4.85% and intervention: 18.22%) and accounting for 30% dropout [61]. The two-sided Z-Test with unpooled variance was used with the significance level of the test is 0.025. Therefore, a sample size of 390 will allow the detection of the change in MVPA or vegetable intake.

All statistical analyses will follow a pre-specified statistical analysis plan guided by our team statistician. Analyses of the primary and secondary outcomes will be conducted according to the intention-to-treat principle. Continuous outcomes at 6-months will be analysed using analysis of covariance (ANCOVA), and categorical outcomes at 6-months will be analysed using log-binomial regression, adjusting for the outcome values at baseline. Planned subgroup analyses will investigate interactions between treatment and subgroups, including categories of age, socioeconomic status and ethnicity, to explore trends for scale up. All tests will be two-sided with a significance level of 0.05. Analyses of the two primary outcomes will be controlled for the family-wise error rate using Holm correction [62]. No multiplicity adjustments will be performed for secondary outcomes as they are exploratory analyses. The analyses will be performed using SAS (V9.4 SAS Institute Inc. Cary NC, USA).

### Ethics approval and consent

Primary ethics approval has been received from the University of Sydney Human Research Ethics Committee (2022/402). Any modifications to the protocol will be submitted to the ethics committee. E-consent will be collected from all participants (and their parents or guardians if < 14-years-old). The sponsor for the study is the University of Sydney and they have no role in the study design, collection, management, analysis and interpretation of data, the writing of findings or submission of findings for publication. The design and conduct and dissemination of the study will be overseen by the Health4Me steering committee (named authors and the Health4Me Team).

## Discussion

Current obesity prevention approaches are ineffective and there are limited programs beyond the school setting available which suit the needs of adolescents, coupled with an escalating mental health crisis for young people in Australia. Positively framed prevention programs which focus on good nutrition and physical activity behaviours can address both critical issues and these programs must be accessible for adolescents. This study aims to provide information and support for lifestyle risk factors by developing and testing a 6-month text message primary prevention program for adolescents in an RCT. It is expected that the results will include improvements or maintenance in primary and secondary outcomes. If effective, this study will inform future translational research to improve the physical and mental health of adolescents and prevent the future development of chronic diseases in adulthood and implementing such a program into healthcare services throughout Australia. Results from the process measures will identify barriers and enablers of widespread implementation of the text message program, with the goal of providing accessible and accurate lifestyle health information and support to adolescents to establish lifelong healthy behaviours.

## Conclusion

This study will test the effectiveness of a 6-month text message intervention to improve and support the physical and mental health outcomes of all adolescents. This study will also provide information on the barriers and enablers of the text message program and ways in which it can be improved. If effective, the results will provide high quality evidence to inform future translational research to scale up the program and embed it within healthcare systems throughout Australia.

## Data Availability

Data sharing is not applicable to this article as no datasets were generated or analysed during the current study.

## Abbreviations

ANCOVA: Analysis of covariance
BMI: Body Mass Index
COVID-19: Coronavirus disease of 2019
GPs: General Practitioners
MVPA: Moderate to Vigorous Physical Activity
RCT: Randomized Controlled Trial
SD: Standard Deviation
SMS: Short Message Service
