# Correction: A healthy lifestyle text message intervention for adolescents: protocol for the Health4Me randomized controlled trial

**DOI:** 10.1186/s12889-024-17698-5

**Published:** 2024-02-20

**Authors:** Rebecca Raeside, Karen Spielman, Sarah Maguire, Seema Mihrshahi, Katharine Steinbeck, Melissa Kang, Liliana Laranjo, Karice Hyun, Julie Redfern, Stephanie R. Partridge, the Health4Me Team

**Affiliations:** 1 Engagement and Co-Design Research Hub, School of Health Sciences, Faculty of Medicine and Health, University of Sydney, Sydney, NSW Australia; 2 Faculty of Medicine and Health, InsideOut Institute, University of Sydney, Sydney, NSW Australia; 3 Sydney Local Health District, Sydney, NSW Australia; 4 Department of Health Sciences, Faculty of Medicine, Health and Human Sciences, Macquarie University, Sydney, NSW Australia; 5 Department of Adolescent Medicine, The Children’s Hospital at Westmead, Sydney, NSW Australia; 6 Specialty of Child and Adolescent Health, Westmead Clinical School, Faculty of Medicine and Health, University of Sydney, Sydney, NSW Australia; 7 General Practice Clinical School, Sydney Medical School, Faculty of Medicine and Health, University of Sydney, Sydney, NSW Australia; 8 Westmead Applied Research Centre, Faculty of Medicine and Health, University of Sydney, Sydney, NSW Australia; 9 Western Sydney Primary Health Network, Sydney, NSW Australia; 10 Department of Cardiology, Concord Repatriation General Hospital, ANZAC Research Institute, Sydney, NSW Australia; 11 The George Institute for Global Health, University of New South Wales, Sydney, NSW Australia; 12 Prevention Research Collaboration, Faculty of Medicine and Health, University of Sydney, Sydney, NSW Australia

**Correction: BMC Public Health 22, 1805 (2022)**

**https://doi.org/10.1186/s12889-022-14183-9**

The original publication of this article contained an incorrect sample size. The incorrect sample size was 330, the correct sample size is 390. The authors have submitted an updated ethical amendment which has been approved. This does not impact the conclusions of the article. The original article has been updated to correct all instances of 330 to 390.
